# Harnessing induced pluripotent stem cells and organoids for disease modeling and precision medicine

**DOI:** 10.1186/s13287-026-04906-9

**Published:** 2026-02-08

**Authors:** Chang-Jin Lee, Yoojun Nam, Yeri Alice Rim, Ji Hyeon Ju

**Affiliations:** 1 Department of Medical Sciences, Graduate School, The Catholic University of Korea, 06591 Seoul, Republic of Korea; 2 CiSTEM Laboratory, Catholic iPSC Research Center, Seoul St. Mary’s Hospital, College of Medicine, The Catholic University of Korea, 06591 Seoul, Republic of Korea; 3 Yipscell Inc, L2 Omnibus Park, Banpo-dearo 222, Seochogu, 06591 Seoul, Republic of Korea; 4 Department of Biohealth Regulatory Science, Sungkyunkwan University, Suwon, South Korea; 5 Division of Rheumatology, Department of Internal Medicine, Seoul St. Mary’s Hospital, Institute of Medical Science, College of Medicine, The Catholic University of Korea, 06591 Seoul, Republic of Korea; 6 Division of Rheumatology, Department of Internal Medicine, Seoul St. Mary’s Hospital, College of Medicine, The Catholic University of Korea, Seoul, 06591 Republic of Korea

**Keywords:** Disease modeling, iPSC, Organoids, Precision medicine, CRISPR screening, Functional genomics

## Abstract

The convergence of CRISPR genome editing, patient-derived organoids, and induced pluripotent stem cells (iPSCs) has reshaped in vitro disease modeling by enabling mechanistic investigations of human pathophysiology within genetically matched, tissue-relevant systems. Together, these technologies provide a synergistic platform for precise manipulation of disease-associated variants and support the generation of isogenic organoid models that reproduce key phenotypic and functional hallmarks across cancer, neurodegenerative, inflammatory, and monogenic disorders. In this review, we highlight how diverse CRISPR modalities—including knock-out, knock-in, CRISPRa/i, and genome-scale screening—have been applied to dissect gene function, model disease progression, and guide therapeutic development using iPSC- and organoid-based systems. We further discuss the application of these platforms in genotype- and phenotype-driven precision medicine, enabling patient stratification, drug-response prediction, and individualized treatment design. We illustrate these convergent applications with representative case studies spanning mechanistic research and early clinical translation. By combining the scalability of genome engineering with the physiological fidelity of organoids, CRISPR-integrated platforms are redefining the frontiers of experimental medicine. These approaches accelerate the discovery of disease mechanisms and actionable therapeutic targets while establishing individualized clinical strategies for complex human diseases. Collectively, they position CRISPR-enabled organoid systems as a foundational infrastructure that bridges genome editing to individualized therapy and supports next-generation precision medicine.

## Introduction

Recent advances in stem cell and organoid technologies have transformed our ability to model human diseases with unprecedented fidelity. Patient-derived induced pluripotent stem cells (iPSCs), when differentiated into lineage-specific cell types, offer genetically matched systems that reflect the patient’s molecular background. In parallel, three-dimensional (3D) organoid cultures recapitulate the spatial architecture, cell-type diversity, and signaling microenvironments of native tissues, overcoming the key limitations of traditional two-dimensional cell cultures and animal models [1]. Together, these technologies provide a foundation for human-relevant in vitro modeling that captures both inter-individual genetic variability and disease-specific phenotypes, thereby supporting precision medicine–oriented experimental systems.

**Fig. 1:**
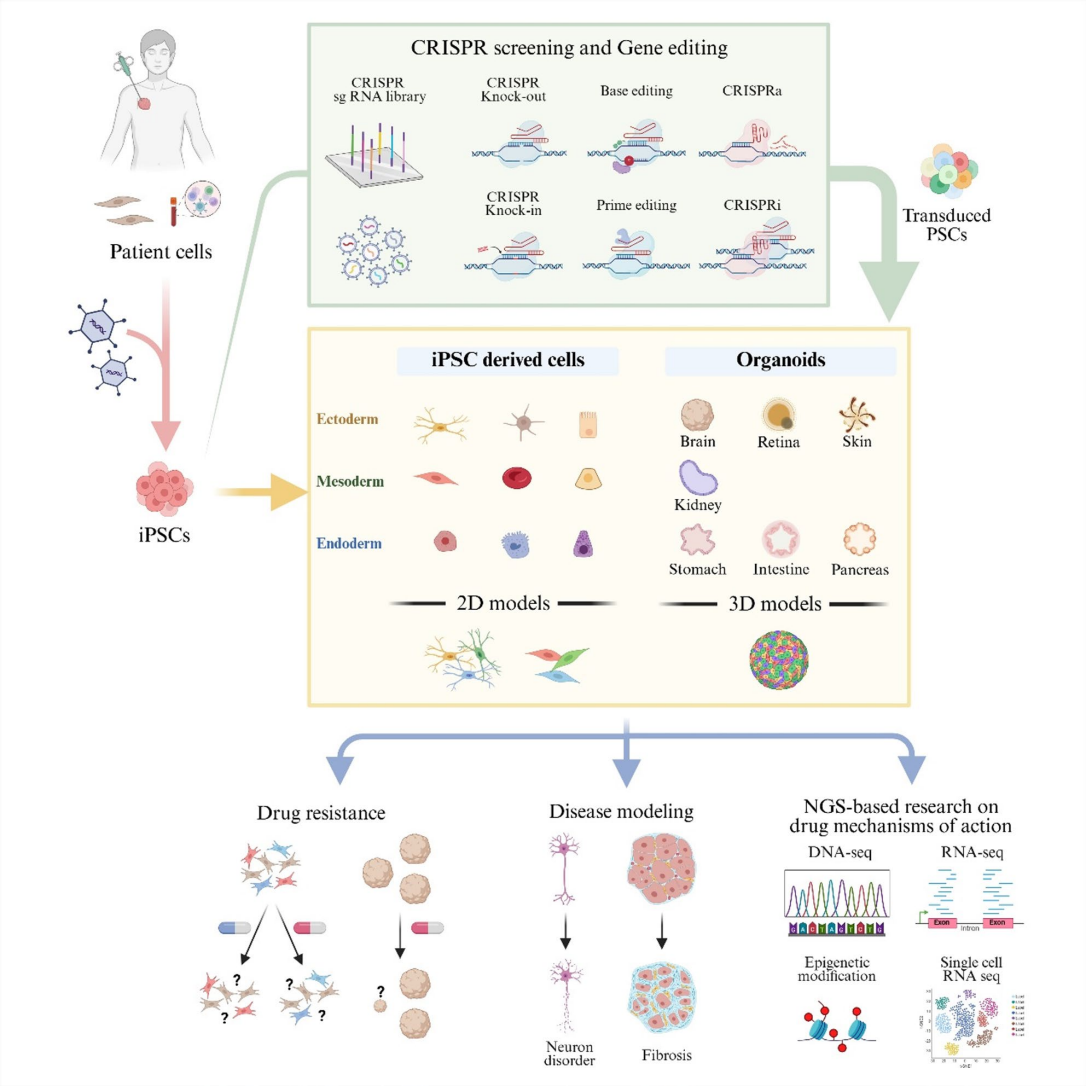
Conceptual framework of CRISPR–iPSC–organoid integration for mechanistic modeling, high-throughput screening, and precision medicine. This figure illustrates how CRISPR-based genome engineering converges with patient-derived iPSC reprogramming and organoid formation to create a multiscale platform for disease modeling and therapeutic discovery. Diverse CRISPR modalities—including knockout, knock-in, base and prime editing, CRISPRa, and CRISPRi—are combined with pooled sgRNA libraries for high-throughput screening, enabling systematic perturbation of gene networks to identify essential pathways, synthetic-lethal interactions, and genotype-specific vulnerabilities. Edited pluripotent stem cells are differentiated into lineage-specific 2D iPSC-derived cells or 3D organoids representing ectodermal, mesodermal, and endodermal tissues, allowing phenotypic consequences of genetic perturbations to be assessed within human-relevant multicellular environments. Readouts span viability, morphology, lineage specification, stress responses, and multi-omic profiling (DNA-seq, RNA-seq, chromatin accessibility, and single-cell transcriptomics). Together, the diagram outlines how pooled and arrayed CRISPR screens integrated with iPSC and organoid systems connect genotype to phenotype at scale, supporting the discovery of disease drivers, resistance mechanisms, and individualized therapeutic targets within a unified precision-medicine pipeline

Importantly, the convergence of CRISPR genome editing, iPSC, and organoid technologies has created a synergistic framework that extends beyond descriptive modeling. Rather than functioning as isolated tools, these platforms interact dynamically—CRISPR provides genetic precision, iPSCs offer a renewable and patient-specific cell source, and organoids supply a physiologically relevant 3D microenvironment. Together, these components forms an iterative, closed-loop system in which genetic perturbations introduced by CRISPR can be functionally interpreted within iPSC-derived and organoid-based tissues, enabling causal mapping from genotype to phenotype and informing individualized therapeutic strategies. Conceptually, Fig. 1 illustrates this synergistic model, highlighting how CRISPR–iPSC–organoid interactions bridge functional genomics and precision medicine by linking variant interpretation, phenotypic reconstruction, and translational decision-making.

Yet, modeling disease complexity requires more than a descriptive replication of tissue structures or markers. Many disorders—especially cancer, neurodegeneration, and immune-related conditions—arise from multigenic alterations and involve dynamic interactions among different cell types and environmental factors. To investigate this complexity, genome engineering tools that allow precise and scalable manipulation of disease-relevant genes are indispensable. CRISPR-based technologies have emerged as a cornerstone for such functional investigation, enabling loss-of-function, gain-of-function, and allele-specific studies directly within human iPSC- and organoid-derived models [2].

The integration of CRISPR into patient-derived platforms has been a major advance in experimental medicine. Using isogenic systems, researchers can dissect the causal effects of individual mutations, perform genome-wide functional screening, and simulate clonal evolution or therapeutic resistance in controlled 3D settings [3]. Moreover, combining CRISPR editing with high-content phenotyping and omics-based profiling (e.g., transcriptomics and proteomics) allows the stratification of patients by genotype–phenotype relationships and the identification of personalized therapeutic vulnerabilities [4]. Such multi-layered integration also supports comparative platform selection—clarifying when iPSC-derived 2D systems are sufficient for mechanistic analysis and when 3D organoid or organ-on-chip models are required to capture higher-order multicellular interactions relevant to clinical translation.

This review highlights the convergence of three domains driving innovation in human disease modeling: (1) patient-derived iPSCs and organoids as genetically faithful, multicellular systems; (2) CRISPR genome editing as a functional genomics engine; and (3) precision medicine approaches that link molecular alterations to individualized therapeutic strategies. Rather than broadly surveying the field, we focus on how these components interact to enable mechanistically informed, clinically translatable disease models. Emphasis is placed on representative case studies, current limitations, and emerging technologies that may bridge remaining gaps [5]. In particular, we incorporate a comparative decision framework, standardized evaluation metrics, and conceptual models to provide a structured, analytically driven perspective on the CRISPR–iPSC–organoid landscape.

## Patient-derived stem cells and organoids as modeling platforms

The development of patient-derived induced pluripotent stem cells (iPSCs) and organoid technologies has revolutionized human disease modeling by providing genetically matched, physiologically relevant systems that recapitulate tissue-specific architecture and molecular complexity. iPSCs, generated from somatic cells using Yamanaka factors, preserve the full genetic landscape of individual patients and can be directed toward multiple lineages, providing a unique window into the cellular and molecular pathophysiology of disease [6]. When assembled into three-dimensional (3D) organoids, these systems self-organize into organ-like structures that capture key multicellular features of native tissues, thereby bridging the gap between monolayer cultures and in vivo biology [7]. Representative disease models derived from patient iPSCs and organoids include cancer, neurodegeneration, and inherited metabolic syndromes. Together, these stem-cell–derived platforms provide a versatile foundation for human disease modeling, supporting both mechanistic investigation and translational application.

To facilitate rational platform selection for experimental or clinical objectives, Table 1 summarizes a practical and conceptual decision matrix outlining the strengths and limitations of key CRISPR–iPSC–organoid modeling strategies. Each row represents a distinct experimental or clinical objective, while the columns guide a stepwise decision process—from defining the research goal to selecting the appropriate platform and genome-editing modality.

**Table 1:** Decision matrix for selecting appropriate stem cell– and organoid-based modeling platforms integrated with CRISPR modalities

Experimental/Clinical Objective	Recommended Modeling Platform	Optimal CRISPR Modality	Primary Strengths	Key Limitations	Representative Disease Areas	Qualitative Performance/Translational Indicators
Causal gene-function analysis	iPSC-derived 2D differentiated cells	Knock-out / Knock-in (HDR, NHEJ)	• High editing efficiency • Homogeneous cell populations ideal for mechanistic dissection	• Minimal microenvironmental cues • Limited multicellular phenotypes	Colorectal cancer models (APC/KRAS), metabolic enzyme deficiencies	• High scalability • Robust editing workflows • Rapid experimental cycles
Pathway dissection & multicellular phenotype validation	3D organoids	CRISPRi / CRISPRa (reversible transcriptional regulation)	• Captures higher-order interactions • No DNA damage; suited for essential/dosage-sensitive genes	• Variable delivery efficiency • Heterogeneous expression across organoids	Neuroinflammatory states, neurodegeneration, epithelial disorders	• Physiologically relevant phenotypes • Enhanced multicellular fidelity
Rare-variant correction & patient-specific therapy testing	iPSC-derived organoids	Base editing/Prime editing	• Precise correction of point mutations • Genotype-matched isogenic control creation	• Delivery and allelic balance challenges • Potential mosaicism	Cystic fibrosis, retinitis pigmentosa, NAFLD variants	• Direct linkage of variant correction to phenotype • High mechanistic resolution
Drug screening & precision-response prediction	Patient-derived tumor organoids (PDTOs) ± immune/stromal co-cultures	Knock-out + multi-omics integration (scRNA-seq, ATAC-seq)	• Retains patient-specific tumor heterogeneity • Strong alignment with clinical therapeutic responses	• Biopsy-dependent • Requires optimized culture conditions	Gastrointestinal cancers, pancreatic cancer, ovarian cancer, glioblastoma	• Predictive of clinical drug response • Suitable for pre-treatment evaluation
Safety assessment & physiological modeling	Organ-on-chip / multi-organoid microphysiological systems	Any modality (KO/KI, base/prime, CRISPRi/a)	• Perfusable interfaces • Simulation of mechanical forces and tissue–tissue communication	• Limited standardization across devices • Low throughput	Cardiotoxicity, lung fibrosis, vascular diseases	• Enhanced physiological realism • Allows dy namic, real-time readouts

For mechanistic gene-function analysis, iPSC-derived 2D differentiated cells combined with knock-out or knock-in editing offer high editing efficiency and scalability, although they lack microenvironmental fidelity [8]. When pathway validation or phenotype testing requires a multicellular context, 3D organoids coupled with CRISPRa/i enable reversible regulation of gene networks without introducing DNA breaks [9].

In contrast, patient-specific therapy testing or rare-mutation correction often benefits from base or prime editing in iPSC-derived organoids, which provide single-nucleotide precision with relatively low off-target activity while continuing to face challenges in delivery and allelic balance [10].

For drug screening and precision-response prediction, tumor organoids (PDTOs) or co-culture models using knock-out editing integrated with omics analysis offer clinically predictive insights while preserving tumor heterogeneity, albeit at higher cost and sample demand. Finally, organ-on-chip multi-organoid systems are emerging for translational physiology and safety assessment, combining perfusable vasculature and immune-vascular interfaces to approximate in vivo conditions, but their broader adoption is limited by non-standardized microfluidic geometries, matrix formulations, and sensor calibration protocols across laboratories [11].

Collectively, Table 1 illustrates how experimental goals dictate platform complexity and how specific CRISPR modalities can be aligned with biological questions to optimize both mechanistic insight and translational relevance. This comparative perspective highlights how each platform occupies distinct yet complementary roles within the precision-medicine workflow.

### Cancer modeling with patient-derived tumor organoids (PDTOs)

Patient-derived tumor organoids (PDTOs), established directly from primary tumors or metastases, retain the intratumoral heterogeneity, mutational signatures, and histoarchitecture of the parental tissue. These models have been successfully generated from a wide spectrum of malignancies, including colorectal, gastric, pancreatic, ovarian, breast, lung, bladder, and glioblastoma [12]. For instance, colorectal cancer PDTOs have demonstrated predictive value for chemoradiation response. Pancreatic cancer PDTOs derived from patients treated with FOLFIRINOX have been used to model adaptive resistance involving reactive oxygen species (ROS), DNA damage, and stem-like transcriptional states [13].

In ovarian cancer, PDTOs allow functional stratification based on homologous recombination deficiency, facilitating response prediction to PARP inhibitors [14]. In glioblastoma, PDTOs co-cultured with autologous CAR-T cells enable the evaluation of antigen-specific immune responses in an immunocompetent context [15]. Additionally, tumor–stroma co-cultures using matched fibroblasts and immune cells have been shown to recapitulate tumor-immune crosstalk, including modulation of checkpoint ligand expression and cytokine secretion profiles, highlighting their use in evaluating immunotherapeutic efficacy and resistance mechanisms.

Beyond drug response prediction, PDTOs have been instrumental in large-scale therapeutic screening. For example, *MTAP* deficiency has been identified as a druggable vulnerability in pancreatic cancer [16]; *SIRT1* overexpression in bladder cancer PDTOs has been linked to poor prognosis and resistance [17]; and *HER2* amplification has guided HER2-targeted therapy in gastric and breast cancer organoids [18]. Co-culture models integrating cancer-associated fibroblasts, autologous immune cells, and endothelial cells further enhance the physiological relevance of PDTOs, enabling studies of immune evasion, stromal remodeling, and anti-angiogenic strategies. Importantly, PDTOs are increasingly being used in early-phase clinical trials to guide personalized treatment strategies and functional validation of molecular diagnostics [19].

In addition, recent applications of organoid systems include modeling responses to novel non-invasive modalities such as Tumor-Treating Fields (TTFs), which use alternating electric fields to inhibit tumor cell division in glioblastoma [20]. Notably, PDTO platforms also serve as validation systems for CRISPR-engineered tumoroids carrying defined oncogenic combinations (e.g., APC/KRAS/TP53/SMAD4), thereby linking genome-engineered hypotheses to clinically anchored functional outputs [21].

Collectively, these studies exemplify how organoid-based cancer models advance both mechanistic understanding and precision-therapy development. Their ability to integrate tumor genetics, microenvironmental interactions, and functional drug responses positions PDTOs as a central decision-support system in translational oncology.

### Modeling neurodegeneration with iPSC-derived organoids

iPSC-derived neural organoids provide access to human neurodevelopment and neurodegeneration in patient-specific contexts. In Alzheimer’s disease (AD), cortical organoids harboring *PSEN1* or *APP* mutations recapitulate amyloid-β plaque accumulation and tau hyperphosphorylation, enabling the study of γ-secretase inhibitors [22]. Midbrain organoids derived from patients with *SNCA* triplication exhibit dopaminergic neuron loss and mitochondrial dysfunction, closely resembling Parkinson’s disease pathology, and serve as platforms for LRRK2 kinase inhibitor screening [23]. These organoids also exhibit impaired mitochondrial biogenesis and synaptic dysfunction, mirroring the early pathophysiological features of PD.

Motor neurons differentiated from iPSCs of patients with ALS carrying *SOD1* mutations show hallmark features, such as cytoplasmic *TDP-43* mislocalization and axonal transport defects, facilitating phenotypic drug screening [24]. Neurons derived from *C9ORF72* repeat-expansion patients demonstrate RNA foci and toxic dipeptide repeat proteins, supporting therapeutic screening with antisense oligonucleotides.

Beyond disease modeling, neural organoids increasingly support precision-medicine applications by enabling patient-specific profiling of pathway-targeted therapies. Compared with 2D neuronal cultures, neural organoids capture multicellular interactions and network-level dysfunction more effectively, thereby enhancing the predictive value of therapeutic assessments in neurodegenerative disease models.

### Modeling monogenic and complex disorders

iPSC-derived organoids have significantly contributed to our understanding of both monogenic and complex diseases. In cystic fibrosis, intestinal organoids homozygous for the *CFTR F508del* mutation exhibit defective chloride transport, which can be quantified using forskolin-induced swelling assays. CRISPR/Cas9-mediated gene correction of *CFTR* restores normal function and validates therapeutic efficacy ex vivo [25].

Retinal organoids generated from patients with *RPGR* mutations replicate photoreceptor degeneration characteristic of retinitis pigmentosa [26]. Pancreatic islet-like organoids derived from iPSCs have been integrated into microfluidic “pancreas-on-a-chip” systems to evaluate β-cell function and insulin secretion in cystic fibrosis–related diabetes [27].

iPSC-derived hepatocyte-like cells from patients with *PNPLA3 I148M* mutations—associated with non-alcoholic fatty liver disease (NAFLD)—display lipid accumulation and endoplasmic reticulum (ER) stress upon fatty acid exposure [28]. Additionally, cortical neurons derived from patients with schizophrenia exhibit reduced dendritic spine density and aberrant synaptic pruning in response to inflammatory cytokines, implicating neuroimmune dysregulation in disease progression [29].

In several of these models, CRISPR-based allele correction or pathway perturbation has begun to clarify genotype-driven mechanisms—for example, restoration of *RPGR* or *PNPLA3* variants—which provides direct evidence linking genetic causality with functional reversibility [28]. A consolidated overview of representative disease models derived from iPSC and organoid systems is provided in Table 2, summarizing key genetic perturbations, phenotypic outputs, and applications across neurological, metabolic, and oncologic conditions.

**Table 2:** Development of disease models using iPSCs and organoids

Technology	Disease	Model Type	Modeling Method	References
Patient-derived iPSCs	Parkinson’s Disease	Dopaminergic Neurons	Patient-derived iPSCs differentiated into dopaminergic neurons to study disease mechanisms	[23]
Patient-derived iPSCs	Alzheimer’s Disease	Neural progenitors	Patient-derived iPSCs to generate neuronal organoids for studying amyloid plaque formation	[22]
Patient-derived iPSCs	Hypertrophic Cardiomyopathy	Cardiomyocyte	Patient-derived iPSCs to model MYH7 mutation-associated hypertrophic cardiomyopathy	[109]
Patient-derived iPSCs	Amyotrophic Lateral Sclerosis	Motor Neurons	Patient-derived iPSCs differentiated into motor neurons to study *SOD1* mutation	[24]
Patient-derived organoids	Cystic Fibrosis	Intestinal Stem Cell Organoids	CRISPR/Cas9-mediated functional repair of the *CFTR* gene, restoring chloride ion transport	[25]
Patient-derived organoids	Retinitis Pigmentosa	Retinal Organoids	Patient-derived retinal organoids to study disease progression and therapeutic screening	[26]
Patient-derived organoids	Cystic Fibrosis	Pancreatic Organoids	Patient-derived pancreatic organoids to study insulin resistance and beta cell dysfunction	[27]
CRISPR Knock-in	Colorectal Cancer	Intestinal Organoids	CRISPR gene editing of *APC*, *SMAD4*, *TP53*, *KRAS*, and *PIK3CA* to model tumor progression	[21]
CRISPR Knock-in	Vascular Anomalies (e.g., Venous Malformations)	Endothelial cells	CRISPR/Cas9 to introduce the *TIE2L914F* mutation in patient-derived iPSCs to study endothelial dysfunction	[37]
CRISPR Knock-out	Liver Cancer	Liver Organoids	CRISPR-engineered liver organoids to study *BAP1* tumor suppressor function	[32]
CRISPR Knock-out	Kidney Disease	Kidney Organoids	CRISPR-mutant kidney organoids derived from human pluripotent epiblast spheroids	[39]
CRISPR Knock-out	Neurodevelopmental Disorders	Cerebral Organoids	CRISPR/Cas9-mediated heterozygous knockout of the *CHD8* gene	[36]
CRISPR Knock-out	Skin Disorders	Human organotypic skin model	CRISPR-Cas9-engineered skin model to study the role of glycosylation in skin development and function	[38]
CRISPRa	Kidney development	Kidney organoids	Investigation of the *HNF4A* gene regulatory network in human kidney organoids to understand proximal tubule differentiation and function.	[44]
CRISPRa/i	Neurological diseases	Microglia	Utilization of a CRISPRi/a platform in iPSC-derived microglia to identify and regulate genes involved in disease states, providing insights into neurological disease mechanisms.	[45]
CRISPRi	Neuroinflammation	Astrocytes	CRISPRi screens in iPSC-derived astrocytes to identify and elucidate regulators of distinct inflammatory reactive states, providing insights into neuroinflammatory pathways.	[47]
CRISPRi	Glioma	Brain organoids	CRISPRi-based screen to identify lncRNA targets modulating radiation response in glioma cells and brain organoids for potential therapeutic intervention.	[48]

Collectively, these examples highlight the versatility of patient-derived stem-cell and organoid systems for modeling diverse disease mechanisms and for establishing preclinical foundations for precision medicine. By capturing the cascade from genomic variation to multicellular dysfunction within controlled isogenic contexts, these systems establish a functional bridge between molecular perturbations and clinically actionable phenotypes.

## CRISPR-based functional modeling using organoids and iPSCs

CRISPR-based gene-editing technologies have revolutionized human disease modeling by enabling precise and customizable modifications of endogenous genes. When applied to patient-derived stem cells and organoid platforms, CRISPR facilitates the generation of disease-specific models that faithfully reproduce genetic, cellular, and tissue-level phenotypes. These models have proven instrumental in uncovering disease mechanisms, identifying therapeutic targets, and guiding the development of precision therapies [30].

Importantly, the integration of CRISPR with iPSC and organoid platforms establishes a unified experimental ecosystem that connects genotype engineering, phenotype manifestation, and therapeutic translation. Conceptually, Fig. 1 depicts this multi-scale workflow—from precise gene editing in pluripotent cells to the emergence of complex tissue-level phenotypes and their application in precision-medicine pipelines. In addition, incorporating pooled sgRNA libraries and high-throughput CRISPR screening into these platforms enables systematic perturbation of hundreds to thousands of targets, allowing quantitative mapping of gene–function relationships within physiologically relevant 3D contexts. This convergence allows direct investigation of causal links between genetic perturbations and emergent disease traits, transforming descriptive modeling into hypothesis-driven discovery.

Collectively, these elements position CRISPR–iPSC–organoid systems as an integrated functional genomics engine that scales from nucleotide-level edits to clinically relevant multicellular physiology.

### CRISPR/Cas9 knock-out and knock-in approaches

CRISPR knock-out and knock-in approaches have enabled researchers to dissect gene functions in a variety of disease contexts. These classic modalities remain the foundation of functional genomics and are most powerful when combined with iPSC- and organoid-based systems that preserve patient-specific genomic backgrounds.

By integrating KO/KI editing with organoid differentiation workflows, investigators can reconstruct disease progression from the earliest mutational events to tissue-level dysfunction, providing mechanistic insight that directly informs therapeutic strategy.

In cancer biology, CRISPR knock-in of oncogenic mutations such as *APC*, *TP53*, *KRAS*, *SMAD4*, and *PIK3CA* into intestinal organoids reconstructs the stepwise transformation from normal epithelium to colorectal adenocarcinoma, enabling the study of mutation-specific tumorigenesis and drug responses [21]. Similarly, in pancreatic organoids, sequential insertion of *KRAS*, *TP53*, *CDKN2A*, and *SMAD4* mutations recapitulates the progression from pancreatic intraepithelial neoplasia (PanIN) to pancreatic ductal adenocarcinoma (PDAC) [31]. In liver cancer models, knock-out of *BAP1* in human liver organoids demonstrates tumor suppressor function, with loss of epithelial polarity and enhanced proliferation typical of hepatocellular carcinoma (HCC) phenotypes [32]. Additionally, *TP53/PTEN* double knock-out in murine liver organoids induces intrahepatic cholangiocarcinoma (ICC)-like transformation, recapitulating the histopathological features of aggressive biliary tract cancers [33].

Beyond oncology, similar CRISPR-based approaches have been extended to non-cancer contexts, allowing mechanistic dissection of neurological and metabolic diseases that were previously inaccessible in vivo. In neurodegenerative disease research, CRISPR-based gene editing has enabled the functional dissection of congenital enzyme deficiencies and mitochondrial pathophysiology using patient-specific organoids. A representative study by Lieberman et al. employed NGLY1-deficient patient-derived iPSCs to generate midbrain organoids and established isogenic CRISPR-corrected controls to investigate disease-relevant phenotypes. The NGLY1-deficient organoids exhibited reduced neuroepithelial expansion, a loss of FOXA2 + progenitors, and delayed dopaminergic differentiation. Multi-omic analyses further revealed compromised oxidative phosphorylation and mitochondrial dysfunction. Notably, CRISPR-mediated correction of the NGLY1 mutation restored neurodevelopmental and metabolic features, validating the causal role of NGLY1 loss-of-function mutations and illustrating the utility of gene-edited isogenic organoids in modeling rare neurodevelopmental disorders [34].

Similarly, in Alzheimer’s disease (AD), CRISPR-mediated knock-out of ABCA7 in human iPSC-derived cortical neurons uncovered a previously uncharacterized mechanism involving disrupted mitochondrial lipid metabolism. ABCA7-deficient neurons showed impaired mitochondrial morphology, increased reactive oxygen species, and dysregulated cholesterol esterification. These abnormalities were rescued by CRISPR-corrected isogenic controls, implicating ABCA7 in maintaining lipid homeostasis and mitochondrial function in neuronal cells [35]. In autism spectrum disorder (ASD), CHD8 haploinsufficiency has been modeled in human cerebral organoids using CRISPR-mediated gene knock-out, recapitulating key features of ASD such as abnormal neural progenitor proliferation, disrupted cortical layer formation, and altered expression of early neurodevelopmental markers (e.g., TBR1, FOXP1). Single-cell RNA-sequencing revealed dysregulation of synaptic signaling pathways and upregulation of Wnt and Notch signaling, suggesting CHD8’s critical role in neurogenesis and cortical circuit formation [36]. These studies collectively demonstrate that KO/KI approaches not only recapitulate disease phenotypes but also enable causal assignment of molecular dysfunction within a controlled isogenic background, which is essential for mechanistic interpretation and target validation.

Beyond neurological disorders, CRISPR modeling has been applied to vascular, dermatological, renal, and metabolic conditions. In vascular malformations, knock-in of the *TIE2^L914F^* gain-of-function mutation in iPSC-derived endothelial cells induces constitutive PI3K/AKT signaling, abnormal vessel pruning, and ectatic vascular structures, recapitulating key clinical features of venous malformation [37]. In dermatology, CRISPR knock-out of *GALNT* family genes in skin organoids has demonstrated the essential role of mucin-type O-glycosylation in epithelial stratification, basal keratinocyte proliferation, and epidermal barrier integrity [38].

In cystic fibrosis, intestinal organoids harboring *CFTR F508del* mutations display defective cAMP-dependent chloride secretion. The forskolin-induced swelling (FIS) assay enables ex vivo functional screening of CFTR modulators, supporting personalized therapeutic approaches. In liver disease, iPSC-derived hepatocyte-like cells carrying the *PNPLA3 I148M* variant exhibit lipid accumulation, oxidative stress, and impaired mitochondrial metabolism, mirroring NAFLD pathology.

The deletion of nephrogenic genes in human pluripotent stem cell–derived kidney organoids has enabled the modeling of congenital anomalies of the kidney and urinary tract, facilitating the study of nephron development and genetic malformations [39]. Moreover, CRISPR knock-out in iPSC-derived cardiomyocytes identified human-specific transporters *SLCO1A2* and *SLCO1B3* that mediate doxorubicin uptake and toxicity, suggesting actionable targets for precision cardioprotection [40]. Finally, CRISPR knock-out in iPSC-derived macrophages uncovered regulators of innate immune pathways, including TLR signaling and autophagy [41].

Taken together, KO/KI studies establish a blueprint for causal inference within isogenic human tissues, enabling reconstruction of multi-hit oncogenic trajectories, validation of rare-disease variants, and scalable mechanistic annotation across organ systems. However, KO/KI approaches are limited when dosage sensitivity, essential-gene function, or fine-grained transcriptional tuning is required—scenarios where base editing or CRISPRa/i is more appropriate.

### CRISPRa and CRISPRi for functional modulation

CRISPRa and CRISPRi extend the functionality of CRISPR by modulating gene expression without altering the underlying DNA sequence [42]. These transcriptional modulation systems serve as precision tools that complement genome-editing approaches by enabling dynamic, reversible regulation of gene networks in iPSC- and organoid-based disease models. Unlike nuclease-dependent CRISPR editing, CRISPRa/i operates on an epigenetic and transcriptional level, offering a safer and less disruptive alternative for targeting dosage-sensitive or essential genes, an advantage particularly emphasized in organoid systems where genomic instability must be minimized.

CRISPRa uses a catalytically inactive Cas9 (dCas9) fused to transcriptional activators, such as VP64 or p300, to enhance gene expression [43]. Unlike knock-in strategies that permanently modify target loci, CRISPRa activates endogenous promoters without altering DNA, thereby preserving chromatin architecture and minimizing clonal variability. This technique has been employed to investigate the HNF4A gene regulatory network in human kidney organoids, providing insights into proximal tubule differentiation and function [44]. Additionally, CRISPRa has been utilized in human iPSC-derived microglia to uncover regulators of disease states and identify key transcriptional networks involved in neurodegenerative disorders such as AD [45]. By enabling tunable gene activation, CRISPRa facilitates the systematic interrogation of developmental and stress-response pathways, bridging mechanistic gene discovery with therapeutic target validation. Recent studies also demonstrate the utility of CRISPRa in multiplex activation screens using pooled sgRNA libraries, enabling parallel upregulation of dozens to hundreds of candidate genes to map lineage-specifying or disease-modifying pathways in organoid systems.

In contrast, CRISPRi utilizes dCas9 fused to transcriptional repressors, such as KRAB, to silence target genes [46]. Functionally, CRISPRi represents the inhibitory counterpart of CRISPRa, allowing stable yet reversible transcriptional repression without introducing double-strand breaks. In iPSC-derived astrocytes, CRISPRi has been employed to identify and elucidate regulators of distinct inflammatory reactive states, providing insights into neuroinflammatory pathways [47]. Additionally, in glioma research, CRISPRi-based screens have been used to identify lncRNA targets that modulate radiation responses in glioma cells and brain organoids, thereby uncovering potential therapeutic interventions [48]. CRISPRi’s high on-target specificity and low mutational footprint make it particularly suitable for essential-gene interrogation, synthetic lethality mapping, and buffering-network analysis within complex 3D tissues.

Together, CRISPRa and CRISPRi form a bidirectional transcriptional control axis: CRISPRa provides gain-of-function, CRISPRi loss-of-function. Both systems can be multiplexed, exhibit low mutational footprints, and maintain stable perturbation profiles within 3D tissues.

In contrast to irreversible KO/KI editing, CRISPRa/i offers fine-grained, tunable, and reversible perturbations—ideal for investigating gene-dosage effects, transient disease states, network buffering, and complex combinatorial pathway logic.

Thus, the CRISPRa/i layer adds regulatory resolution that KO/KI alone cannot achieve, completing the functional-modulation spectrum required for a comprehensive precision-medicine toolkit.

### Advancing disease modeling and precision medicine through high-throughput CRISPR screening and multi-omics integration

High-throughput CRISPR screening enables systematic and quantitative identification of disease-driving genes, pathway dependencies, and synthetic-lethal interactions across iPSC- and organoid-derived models. In colorectal cancer, combinatorial knock-out of TGFBR2 and SMAD4 revealed synthetic lethality under TGF-β signaling pressure, highlighting strategies for targeting resistance in microsatellite-stable tumors [49]. These scalable perturbation platforms now permit multi-dimensional mapping of gene-function networks under defined microenvironmental or cytokine conditions, enabling functional interrogation of context-specific vulnerabilities and enhancing mechanistic resolution in disease modeling [50].

Integrating CRISPR screening with multi-omics approaches, particularly single-cell RNA sequencing (scRNA-seq), ATAC-seq, and spatial transcriptomics, enables single-cell–resolved reconstruction of perturbed regulatory hierarchies. For example, in liver organoids, the OSCAR platform profiled more than 80,000 cells across 246 CRISPR perturbations, combining transcriptomic and lineage-tracing signatures to identify *Fos* and *Ubr5* as regulators of hepatocyte specification and metabolic zonation [51]. In melanoma, CRISPR-guided base-editing coupled with scRNA-seq delineated adaptive transcriptional reprogramming and mapped SOX10–MITF regulatory axes underlying tumor plasticity and immune evasion [52].

Such integrative approaches reveal non-linear regulatory rewiring that cannot be captured by bulk analyses and simultaneously expose several current bottlenecks, including culture-to-culture variability, editing-efficiency heterogeneity, and challenges in harmonizing multi-omic datasets across laboratories [53]. In addition, incorporating basic translational performance metrics—such as predictive concordance, assay turnaround time, and culture success rates—will further clarify the clinical utility of CRISPR-integrated organoid screens. Emerging consortium-level reporting frameworks and FAIR-like standards for perturbation datasets are expected to enhance reproducibility and support future regulatory acceptance [54, 55].

Machine-learning algorithms further expand these multi-omic datasets into predictive frameworks. Supervised and graph-based learning models improve sgRNA design by modeling sequence–efficacy relationships, integrate high-dimensional omics features into latent regulatory manifolds, and prioritize candidate biomarkers through feature attribution and network centrality analyses. In papillary renal cell carcinoma, ML-assisted CRISPR screening uncovered *SERPINH1* as a prognostic determinant linked to extracellular matrix remodeling and drug response heterogeneity [56–58]. Recent work has also shown that generative modeling and hybrid mechanistic–statistical models can simulate perturbation outcomes across organoid differentiation trajectories, offering an in silico complement to experimental screens and enabling hypothesis filtering before wet-lab validation [59]. Beyond target identification, AI-driven models now support cross-laboratory normalization of CRISPR screens, probabilistic estimation of therapeutic response curves, and adaptive optimization of editing strategies [60].

To ensure reproducibility and clinical readiness, standardized computational pipelines and harmonized data schemas are essential. Containerized workflows with version-controlled reference genomes, batch-correction matrices, and cross-modal feature alignment can minimize algorithmic drift. Furthermore, emerging guidelines emphasize the need for transparent reporting of sgRNA design criteria, perturbation efficiency metrics, and multi-omic integration steps to support inter-study comparability [61]. In parallel, interoperable data-sharing frameworks and model cards that document dataset provenance, hyperparameter configuration, and performance benchmarks will be indispensable for regulatory transparency and external validation.

Collectively, the convergence of high-throughput CRISPR screening, multi-omics integration, and AI-assisted analytics provides a scalable, reproducible engine for precision-medicine discovery—linking molecular perturbations to clinically actionable phenotypes with interpretability and cross-platform robustness. This integrated workflow increasingly functions as a translational scaffold that connects mechanistic gene perturbation to clinically relevant endpoints, thereby accelerating the development of individualized therapeutic strategies across diverse disease contexts.

## Genotype- and phenotype-based precision medicine

Organoids and stem cell-derived models have redefined the field of precision medicine by enabling the generation of patient-specific disease systems that directly connect genetic variation to phenotypic outcome and clinical response. These integrated models extend beyond descriptive replication to establish a functional bridge between molecular genetics, cellular physiology, and individualized therapy design.

**Fig. 2:**
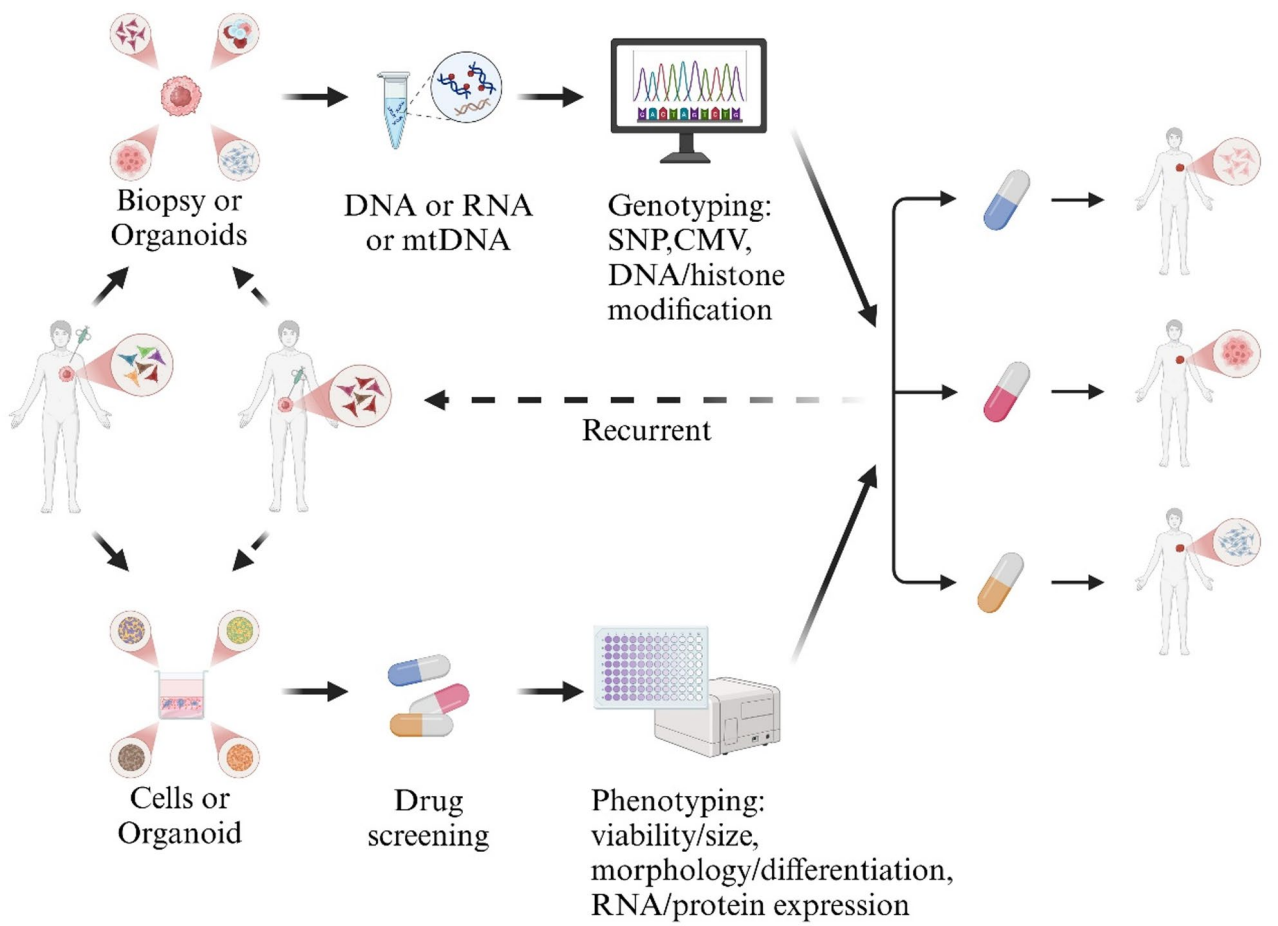
Integrated workflow linking genotype- and phenotype-based precision-medicine strategies using patient-derived stem cells and organoids. This figure presents a translational pipeline in which patient biopsies or organoids undergo genomic profiling—including single-nucleotide variants, copy-number changes, mitochondrial DNA alterations, and epigenetic or histone modifications—to identify mutation-specific vulnerabilities and guide therapeutic selection. In parallel, patient-derived cells or organoids are subjected to phenotypic assays such as drug screening, viability and size measurements, differentiation and morphology profiling, and RNA or protein expression analyses, enabling functional characterization of therapeutic responses. Iterative cycles of genotyping, perturbation testing, and phenotyping establish a recurrent decision-making loop that refines individualized treatment strategies. By integrating molecular determinants with functional cellular behavior, this workflow demonstrates how genotype- and phenotype-derived information converge to improve patient stratification, predict drug efficacy, and support clinical implementation of personalized therapies in monogenic, complex, and heterogeneous diseases

These advanced models allow researchers to design tailored therapeutic strategies guided by an individual’s genotype (genetic composition) and phenotype (observable cellular and tissue traits) (Fig. 2) [62]. By unifying these dimensions, researchers can delineate causal pathways, stratify patient subtypes, and link experimental findings to clinical endpoints in both monogenic and complex multifactorial diseases such as cancer.

### Genotype-driven precision medicine using organoids and stem cells

Genotype-guided models have been pivotal in elucidating mutation-specific mechanisms and testing targeted interventions. In cystic fibrosis, caused primarily by the *F508del* mutation in the *CFTR* gene, patient-derived intestinal organoids enable quantitative functional assays of CFTR activity and allow the testing of modulator drugs such as Ivacaftor, Lumacaftor, and Trikafta, which restore chloride-channel conductance and show strong correlation with clinical response metrics [25]. In hypertrophic cardiomyopathy (HCM), iPSC-derived cardiomyocytes carrying *MYH7* mutations exhibit increased sarcomere tension and impaired ATP utilization, clarifying mutation-specific bioenergetic defects. These models have facilitated rational drug development, including the design of Mavacamten, a myosin ATPase inhibitor that reduces sarcomere contractility and normalizes myocardial output [63, 64].

In colorectal cancer, organoids engineered with *APC*, *KRAS*, *TP53*, and *SMAD4* mutations model stepwise tumorigenesis and enable genotype-specific drug screening. Organoids harboring *KRAS* or *APC* mutations identified synthetic lethality via combined EGFR and MEK inhibition, providing a mechanistic rationale for clinical polytherapy design [65]. Similarly, gastric and pancreatic tumor organoids stratified by *ERBB2* amplification or *BRCA* mutation exhibit differential sensitivity to HER2-targeted or PARP-inhibitor therapies [66–68].

In neurodegenerative disorders, patient-specific iPSC-derived organoids have advanced the mechanistic dissection of genotype–phenotype coupling. iPSCs carrying *SNCA* or *LRRK2* mutations differentiate into dopaminergic neurons that display mitochondrial dysfunction and α-synuclein aggregation, providing direct assay platforms for LRRK2 kinase inhibitors [69, 70]. Alzheimer’s disease organoids harboring *PSEN1* or *APP* mutations reproduce amyloid-β deposition and enable γ-secretase inhibitor testing [71–73]. In addition, integrative genomic and transcriptomic studies have elucidated key regulatory networks and molecular subtypes associated with disease heterogeneity, revealing distinct gene expression profiles that may serve as patient-specific therapeutic targets [74]. One such example is the identification of *IDE* (insulin degrading enzyme) regulatory variants that influence Alzheimer’s disease risk by modulating *IDE* expression in the brain, as demonstrated by integrative expression and genome-wide association analyses [75]. These findings underscore the value multi-omics–driven, genotype-informed precision strategies in identifying individualized treatment approaches for Alzheimer’s disease.

Beyond the central nervous system (CNS), *RPGR*-mutant retinal organoids have facilitated CRISPR-based gene correction and pharmacological screening for retinitis pigmentosa [76]. In metabolic disorders such as NAFLD, liver organoids engineered with *PNPLA3* or *TM6SF2* variants are used to test FXR and PPAR agonists, providing a platform for genotype-dependent lipid metabolism intervention [77, 78].

### Phenotype-driven precision medicine using organoids and stem cells

While genotype defines the molecular blueprint, phenotype-based models capture emergent disease features shaped by epigenetic, environmental, and multicellular interactions. Patient-derived organoids faithfully reproduce tissue morphology, dynamic signaling, and drug-response heterogeneity in vitro, allowing real-time profiling and functional validation of therapeutic responses.

In oncology, organoids derived from pancreatic and gastric tumors display subtype-specific phenotypes, such as basal-like versus classical morphology in pancreatic cancer, which correlate with the response to FOLFIRINOX or gemcitabine [79, 80]. Ovarian cancer organoids classified by homologous recombination deficiency (HRD) predict response to PARP inhibitors such as Olaparib with > 80% ex vivo–to–clinical concordance, demonstrating the predictive validity of phenotype-driven platforms [81].

In infectious diseases, brain organoids have revealed ZIKA virus–induced phenotypes, such as cortical thinning and progenitor cell apoptosis. These models enabled the repurposing of Sofosbuvir to inhibit viral replication in human neural progenitor cells [82, 83]. Cardiomyocytes derived from iPSCs of patients with long QT syndrome or dilated cardiomyopathy display distinct electrophysiological signatures—delayed repolarization and reduced contractile amplitude—that serve as quantitative phenotypic readouts for β-blocker or ion-channel modulator testing [84–86].

### Integrating genotype and phenotype for translational readiness

Integrating genotype- and phenotype-based modeling provides a mechanistic framework for linking molecular perturbations to emergent biological function. Rather than viewing genotype and phenotype as isolated endpoints, this integration treats them as interdependent layers within a dynamic regulatory hierarchy, where information flows bidirectionally between molecular networks and system-level outcomes. In patient-derived systems, this bidirectional information flow becomes experimentally tractable because genome engineering can be coupled with quantitative phenotyping across developmental, metabolic, and tissue-level readouts.

At the experimental level, organoid and iPSC-based platforms serve as intermediate translators between genomic variation and functional phenotypes. Functional reconstitution of patient-derived variants has revealed that pathogenic mutations often reshape developmental and metabolic pathways via non-linear feedback within signaling networks rather than through simple loss or gain of function [87]. In β-catenin-related neurodevelopmental disorders, for example, reconstituted mutations within iPSC-derived organoids demonstrate graded alterations in Wnt-responsive transcriptional programs and neuronal patterning consistent with patient-specific phenotypic spectra. Such approaches illustrate how controlled genetic perturbation can elucidate the causal path from genomic variation to complex tissue-level outcomes and enable more refined stratification of molecular subtypes based on differential pathway engagement.

At the systems scale, emerging frameworks describe genotype-to-phenotype translation as a cascade across a hierarchy of cellular subsystems, where perturbations in gene regulatory circuits propagate through metabolic and signaling networks to yield coordinated phenotypic states [88]. This view emphasizes that phenotypic stability arises from network-level compensation and feedback, and that disease manifests when these buffering mechanisms are exceeded. Within this hierarchical context, organoid models provide an empirical means to quantify propagation of perturbations and identify control nodes whose modulation can restore functional homeostasis, for example, via rescue phenotyping in isogenic pairs or network-rewiring analysis in single-cell datasets.

Analytically, multi-scale data integration has become essential for capturing these cross-level interactions. Single-cell transcriptomic, chromatin-accessibility, and proteomic datasets are now merged through graph-based and probabilistic learning approaches to map latent relationships between genetic variation and phenotypic output [89]. Explainable AI frameworks further enhance interpretability by linking molecular features to emergent functional states and predictive biomarkers while also quantifying how specific genomic edits influence regulatory trajectories during organoid differentiation.

Quantitative benchmarks such as predictive concordance (70–90%), culture-success rate (70–85%), and assay turnaround time (10–21 days) are increasingly used to assess reproducibility and translational readiness in organoid-based precision-medicine pipelines [90]. Recent studies in gastrointestinal and colorectal tumor organoids have reported approximately 80% concordance between ex-vivo drug responses and clinical outcomes [91], with culture-success rates exceeding 70% under optimized media conditions [92], and streamlined drug-screening pipelines achieving turnaround times of 4–6 weeks that are clinically actionable [93]. These metrics underscore the growing maturity of organoid-based assays as translationally deployable precision-medicine tools.

Conceptually, this integration forms a translation-ready knowledge layer that underpins clinical implementation without overlapping its regulatory domain. Recent analyses of genomic-driven precision-medicine infrastructure highlight the importance of technological readiness—standardized bioinformatics pipelines, validated cellular reference models, and interoperable data frameworks—to support reproducibility and scalable deployment [94]. As these infrastructures continue to evolve, genotype–phenotype integration is expected to support prospective patient stratification, adaptive clinical-trial design, and real-time therapeutic decision-making across heterogeneous disease populations. Together, these advances transform genotype-phenotype integration from a descriptive exercise into a predictive, mechanistically grounded foundation for precision medicine.

## Case studies and translational application

Patient-derived organoids (PDOs) are transforming precision medicine by providing patient-specific platforms that preserve key aspects of tissue architecture, genetics, and function. Clinically, PDOs are applied in various contexts, ranging from mechanistic disease modeling to personalized treatment guidance. Beyond serving as ex vivo surrogates, PDOs enable functional assessment of therapeutic responses that complement genomic profiling and address biological questions not accessible through conventional sequencing alone. Recently, organoid technologies have been integrated into clinical workflows, including patient stratification, drug sensitivity testing, and personalized therapy selection. Ongoing clinical trials utilizing organoid models across various disease areas are summarized in Table 3. Collectively, these studies position PDOs within a translational continuum that links molecular characterization with functional phenotyping and downstream therapeutic refinement. As outlined in PDO-based assays are increasingly situated within a translational workflow that integrates molecular profiling, functional phenotyping, and therapy optimization.

**Table 3:** Organoid-Based clinical trials for disease modeling and precision therapy

Disease Area	Organoid Source	Clinical Use/Outcome	Trial No.
Breast Cancer	Tumor PDOs	Personalized drug screening based on genomic profiling	NCT04450706
Breast Cancer	Tumor PDOs (TRIPLEX trial)	Chemotherapy prediction using iPDTOs with autologous immune cells	NCT05404321
Colon Cancer	Tumor organoids	Effectiveness of imatinib as pre-operative therapy	NCT02685046
Colorectal Cancer	Tumor PDOs	Validation of organoid-based diagnostics for chemoradiation sensitivity	NCT03577808
Colorectal Cancer	Tumor PDOs	Response stratification to chemotherapy and *EGFR* inhibitors	NCT05883683
Cystic Fibrosis	Intestinal organoids	Personalized *CFTR* modulator response prediction	NTR7520
Endometrial Cancer	Endometrial organoids	Prediction of malignancy evolution and recurrence risk	NCT06841653
Esophageal Cancer	Tumor organoids	Prediction model development for chemoradiotherapy response	NCT03081988
Esophageal Cancer	Tumor PDOs	Prediction of chemoradiation response	NCT03283527
Food Allergy	Intestinal organoids	Response to allergen insult in food allergy screening	NCT05056610 / NCT05259826
Gastric/Esophageal Cancer	Tumor organoids	Organoid response correlation with systemic chemotherapy outcomes	NCT03429816
Hepatocellular Carcinoma	CTC organoids	Organoid culture of CTCs for genomic characterization and prognosis	NCT05242237
Infertility	Endometrial organoids	Implantation failure in recurrent pregnancy loss	NCT04939064
Inflammatory Bowel disease	Intestinal organoids	Evaluation of inflammatory and tumor markers to characterize disease progression	NCT02874365
Kidney Disease	iPSC-derived kidney organoids	Transcriptional profiling of ciliopathy	NCT04874909
Lung Cancer	Tumor PDOs	Radiation sensitivity profiling using hypoxia-activated prodrugs	NCT04859166
Metastatic Pancreatic Cancer	Tumor organoids	Predicting drug response using organoids and organotypic culture systems	NCT03500068
Pancreatic Cancer	Tumor organoids	Prediction of treatment response based on organoid reactivity and genomic analysis	NCT04777604
Pancreatic Cancer	Intestinal organoids	Effect of nutritional formula on gut barrier and microbiota	NCT06852014
Pancreatic Cancer Resectable	Tumor organoids	Post-surgical prognosis and adjuvant therapy response prediction using PDOs	NCT04736043
Pancreatic Ductal Adenocarcinoma	Tumor organoids	Validation of biopsy-derived organoids for drug response and tumor marker expression	NCT06666803
Psychiatric Disorders	iPSC-derived brain organoids	Modeling of genetic variants related to psychiatric conditions	NCT05480826
Soft Tissue Sarcoma	PDO/PDX/3D culture	Preclinical stratification and drug testing platform	NCT02910895
Solid Tumors	Tumor organoids	NGS-guided therapy stratification using biopsy-derived data	NCT01904916
Xerostomia	Salivary gland organoids	Restoration of salivary function post-radiotherapy	NCT04593589

In disease prognosis, PDOs have been applied across diverse disease areas to assess individual pathophysiology. Intestinal organoids from patients with inflammatory bowel disease (IBD) are being analyzed in a clinical trial (NCT02874365) to characterize inflammatory and tumor markers related to disease progression in Crohn’s disease and ulcerative colitis. Similarly, food allergy diagnostics are being explored using intestinal organoids exposed to allergens (NCT05056610 and NCT05259826). iPSC-derived kidney organoids are employed for transcriptional profiling in ciliopathy (NCT04874909), while endometrial organoids are used to study recurrent pregnancy loss (NCT04939064). Psychiatric disorders have been modeled using brain organoids derived from iPSCs to evaluate the impact of genetic variants (NCT05480826). In oncology, organoid-based matrix invasion and viability assays are being used to assess prognostic applications and tumor aggressiveness. Collectively, these prognosis-oriented applications demonstrate the capacity of PDOs to capture patient-specific variation in disease progression and microenvironmental responses, thereby supporting early risk stratification and mechanistic interpretation of clinical heterogeneity.

For therapeutic guidance, colorectal cancer organoids have been extensively studied in multiple trials (NCT05883683, NCT05832398, NCT05352165, and NCT04220242) to predict responses to chemotherapy and EGFR inhibitors. Organoids have also been used to assess chemoradiation sensitivity before surgery for rectal cancer (NCT03577808). In breast cancer, trials include personalized drug screening using genomic profiling (NCT04450706) and the TRIPLEX trial (NCT05404321), which evaluates chemotherapeutic responses using co-cultured tumor organoids and autologous immune cells. In pancreatic and esophageal cancers, PDOs are being tested to stratify chemotherapeutic efficacy (e.g., NCT03283527), whereas in lung cancer, biobanked organoids are used for radiation sensitivity prediction using hypoxia-activated prodrugs (NCT04859166). Across these disease contexts, several independent studies have reported 70–90% concordance between organoid-derived drug sensitivity profiles and patient responses, underscoring their emerging utility as clinically informative platforms for pre-treatment therapeutic evaluation.

Beyond cancer, therapeutic organoid models have shown value in monogenic diseases. In cystic fibrosis, rectal organoids are being used in the HIT-CF trial (NTR7520) to guide CFTR modulator therapy for patients with rare mutations. This ex vivo assay predicts personalized drug responses and supports the expanded access to targeted treatments. Salivary gland organoids have been used to restore gland function after radiotherapy in patients with xerostomia (NCT04593589). These examples highlight how organoid-based functional assays extend precision-medicine strategies to monogenic and rare diseases, where therapeutic options are limited and mutational spectra are highly heterogeneous.

Collectively, these case studies illustrate how PDOs operate as integrative platforms that unify mechanistic modeling, diagnostic stratification, and therapeutic evaluation. As clinical adoption progresses, PDO-guided diagnostics and functional testing pipelines are expected not only to refine patient stratification but also to provide actionable evidence for therapy selection, thereby strengthening the translational bridge between experimental modeling and individualized clinical care.

## Challenges and future directions

Despite significant progress enabled by CRISPR genome editing and patient-derived organoid technologies, multiple technical and translational limitations continue to constrain their broad implementation in preclinical and clinical settings. A central challenge is the presence of multi-scale fidelity gaps—ranging from cellular composition to microenvironmental cues and systems-level physiology—that limit the ability of current models to recapitulate in vivo biology. Most organoid systems lack perfusable vasculature, functional immune components, and dynamic extracellular matrix (ECM) remodeling. These deficiencies impair the modeling of key processes such as immune–tumor interactions, fibrosis-associated matrix stiffening, and regenerative responses. For example, tumor organoids co-cultured with tumor-infiltrating lymphocytes (TILs) or myeloid-derived suppressor cells (MDSCs) often fail to sustain immune activity due to cytokine depletion and exhaustion [95], while fibrosis organoids seldom incorporate viscoelastic ECM gradients that drive fibroblast activation and therapeutic resistance [96]. Additionally, batch-to-batch variability in ECM products such as Matrigel introduces uncontrolled variation in stiffness and proteomic composition, complicating genotype–phenotype interpretation and limiting reproducibility. These fidelity gaps underscore the need for chemically defined and regulatory-compatible ECM alternatives.

Bioengineered microfluidic systems offer a promising route to address several of these limitations. Platforms incorporating tunable hydrogels, defined mechanical properties, and spatial cytokine gradients improve physiological relevance and enable controlled interrogation of mechanotransduction pathways [97, 98]. Lung-on-chip devices have been used to model TGF-β–mediated fibrotic remodeling under cyclic strain that mimics breathing [99], while cancer-on-chip platforms integrating endothelialized channels and stromal compartments have enabled real-time analysis of PD-L1 regulation during immune checkpoint blockade [100]. However, most microphysiological systems remain custom-built, low-throughput, and insufficiently standardized in geometry, flow parameters, and calibration procedures, limiting cross-study comparability and translational adoption. Recent guidance from regulatory agencies such as the FDA and EMA highlights the need for traceable assay conditions, validated materials, and standardized device characterization—criteria that current academic prototypes only partially fulfill.

Another limitation is the insufficient integration of real-time, multiparametric biosensing within organoid and organ-on-chip systems. While sensors for transepithelial electrical resistance (TEER) and oxygen monitoring exist, few platforms support simultaneous and longitudinal measurement of cytokine secretion, ECM remodeling, and metabolic fluxes at single-cell resolution [101]. This constrains the study of dynamic disease processes and limits the interpretability of CRISPR perturbation studies, where transient or adaptive phenotypes may go undetected. Recent advances—including scalable secretome multiplexing and barcoded organoid perturbation assays—provide potential solutions but remain early in development and require standardized protocols before they can support clinical decision-making.

Reproducibility and standardization remain major obstacles. Variability in donor cell sources, ECM composition, media formulations, and genome-editing workflows introduces substantial experimental heterogeneity. Emerging initiatives that provide modular organoid-chip systems, harmonized fluidic controllers, and cryopreserved isogenic cell banks aim to mitigate such variability [5]. Complementary biophysical characterization methods—including atomic force microscopy for stiffness profiling and mass spectrometry–based ECM proteomics—will be essential for benchmarking and quality control [102]. Establishing minimum reporting standards for organoid–CRISPR studies—covering donor metadata, ECM mechanical properties, editing fidelity, cytokine-stability metrics, and standardized multi-omics formats—will further enhance reproducibility and regulatory acceptance. International biobanking frameworks, including OECD guidelines, additionally emphasize the importance of long-term governance, consent policies, and data privacy for large-scale organoid repositories.

As organoid systems increase in complexity, the resulting multi-omic and imaging-based datasets require sophisticated computational frameworks capable of managing high-dimensional, time-resolved data. While machine-learning models have improved sgRNA design and drug-response prediction, few pipelines are optimized for organoid-specific modalities such as lineage-traced single-cell trajectories, spatial transcriptomics, or integrated biosensor outputs [103]. Cross-modal data integration platforms that unify imaging-based phenotypes with perturbation signatures and metabolic dynamics will be essential for translating organoid datasets into clinically actionable insights. Computational workflows will also need to incorporate transparency, traceability, and external validation to meet emerging regulatory expectations. Early clinical studies demonstrating the predictive accuracy of organoid transcriptomic signatures—such as scRNA-seq–based classifiers for FOLFOX response in colorectal cancer—highlight the translational potential of integrated analytics [104].

Scaling organoid technologies for clinical deployment presents additional challenges. Although early-phase trials demonstrate the promise of patient-derived organoids (PDOs) in guiding treatment decisions, obstacles persist, including genetic stability, manufacturing reproducibility, and the lack of clinically validated quality metrics [105]. Current organoid models also lack systemic physiological features such as organ–organ communication, pharmacokinetic profiles, and immune toxicity, limiting their predictive value. Multi-organ-on-chip systems that emulate circulatory coupling and metabolic exchange may help address these gaps [106]. In parallel, issues related to equity, patient consent governance, long-term biobank stewardship, and data privacy must be addressed as organoid-based diagnostics transition toward routine clinical use.

Looking ahead, the convergence of genome engineering, tissue engineering, and computational modeling is positioned to generate highly integrative disease platforms with enhanced predictive capacity. High-fidelity Cas variants, inducible CRISPR systems, and non-viral delivery strategies may improve editing precision and temporal control, while engineered ECMs that emulate tissue-specific biochemical and mechanical properties are likely to increase physiological relevance [107]. Ultimately, scalable and GMP-compliant organoid platforms equipped with real-time biosensing, harmonized analytics, and validated performance metrics will be essential for transitioning these technologies from research tools into clinically deployable precision-medicine infrastructure [108]. Sustained progress will require coordinated multi-institutional harmonization efforts, interoperable organoid biobanks with transparent governance, and regulatory frameworks capable of supporting the clinical qualification of organoid-based assays.

## Conclusions

The convergence of CRISPR-based genome editing and patient-derived organoid technologies has reshaped the landscape of human disease modeling, providing genetically accurate, mechanistically tractable, and clinically relevant platforms. Unlike traditional in vitro systems, these models enable the functional interrogation of disease-associated variants within a native-like multicellular and three-dimensional context. Through isogenic comparisons and genome-wide perturbation screens, researchers can now delineate causal gene-function relationships, uncover synthetic lethal interactions, and stratify patients based on genotype–phenotype correlations.

Importantly, the integration of CRISPR technology has extended organoid utility from descriptive phenotyping to hypothesis-driven discovery, allowing the dissection of developmental pathways, tumor evolution, and inflammatory signaling with unprecedented specificity. High-throughput perturbation approaches, when combined with multi-omics and AI-assisted analytics, are accelerating the identification of actionable targets and resistance mechanisms across cancer, neurodegenerative, metabolic, and rare genetic diseases and are increasingly being embedded into early translational decision-making pipelines.

Nonetheless, key challenges persist—including the incomplete recapitulation of physiological microenvironments, variability in organoid generation protocols, and limitations in real-time biosensing and computational integration. Overcoming these hurdles will require coordinated advances in bioengineering, gene delivery, and data science, as well as the establishment of standardized, regulatory-compliant workflows for clinical deployment including minimum reporting standards, validated reference organoid lines, interoperable data formats, and transparent benchmarking frameworks. Equally critical will be the development of clear regulatory pathways for organoid-based diagnostics and functional assays, informed by emerging FDA/EMA guidance on microphysiological systems and genome-edited cell platforms.

As organoid systems become increasingly integrated with immune, vascular, and microbiome components—and embedded within dynamic organ-on-a-chip frameworks—their value as predictive, personalized platforms will only grow. In parallel, advances in synthetic biomaterials, high-fidelity Cas variants, and non-viral precision delivery systems are expected to further enhance editing stability and phenotypic robustness. Furthermore, community-wide adoption of open-access model repositories, federated data-sharing infrastructures, and harmonized informatic pipelines will be essential to achieve reproducibility, equity, and global scalability of organoid-enabled precision medicine. Attention to ethical governance, biobank stewardship, and patient-consent frameworks will also be required to ensure responsible and equitable deployment.

Together, these technologies are ushering in a new era of functional precision medicine, enabling mechanistically guided diagnosis, individualized therapy optimization, and ultimately, improved outcomes for patients with complex and heterogeneous diseases while simultaneously laying the translational foundation for regulatory-ready, clinically deployable organoid platforms in the coming decade.

## Data Availability

All data (or sources thereof) relevant to this study are included in the article, and further inquiries can be directed to the corresponding author.
